# LINC01106 post-transcriptionally regulates ELK3 and HOXD8 to promote bladder cancer progression

**DOI:** 10.1038/s41419-020-03236-9

**Published:** 2020-12-12

**Authors:** Liwei Meng, Zhaoquan Xing, Zhaoxin Guo, Zhaoxu Liu

**Affiliations:** Qilu Hospital of Shandong University, Jinan, 250000 Shandong Province China

**Keywords:** Bladder cancer, Cell biology

## Abstract

Bladder cancer (BCa) is a kind of common urogenital malignancy worldwide. Emerging evidence indicated that long noncoding RNAs (lncRNAs) play critical roles in the progression of BCa. In this study, we discovered a novel lncRNA LINC01116 whose expression increased with stages in BCa patients and closely related to the survival rate of BCa patients. However, the molecular mechanism dictating the role of LINC01116 in BCa has not been well elucidated so far. In our study, we detected that the expression of LINC01116 was boosted in BCa cells. Moreover, the results of a series of functional assays showed that LINC01116 knockdown suppressed the proliferation, migration, and invasion of BCa cells. Thereafter, GEPIA indicated the closest correlation of LINC01116 with two protein-coding genes, ELK3 and HOXD8. Interestingly, LINC01116 was mainly a cytoplasmic lncRNA in BCa cells, and it could modulate ELK3 and HOXD8 at post-transcriptional level. Mechanically, LINC01116 increased the expression of ELK3 by adsorbing miR-3612, and also stabilized HOXD8 mRNA by binding with DKC1. Rescue experiments further demonstrated that the restraining influence of LINC01116 knockdown on the progression of BCa, was partly rescued by ELK3 promotion, but absolutely reversed by the co-enhancement of ELK3 and HOXD8. More intriguingly, HOXD8 acted as a transcription factor to activate LINC01116 in BCa. In conclusion, HOXD8-enhanced LINC01116 contributes to the progression of BCa via targeting ELK3 and HOXD8, which might provide new targets for treating patients with BCa.

## Introduction

Bladder cancer (BCa) is one of the most prevalent urogenital malignancies all over the world^1,2^. The high recurrence and mortality of BCa showed an increasing trend year by year^3,4^. Many factors participate in the tumorigenesis and progression of BCa, such as smoking, chemicals, and abnormal expression of genes^5^. In spite of great advancements in the treatment of BCa, the overall survival of high-risk patients is still not optimistic^6,7^. As far as we know, an increasing number of evidence has reported the importance of abnormally expressed genes in BCa progression^8,9^. On this basis, targeted therapy has developed as a new road for treating patients with BCa, especially those with advanced BCa^10^. Therefore, it is necessary to find more effective therapeutic targets for BCa treatment.

Long noncoding RNAs (lncRNAs) are a kind of RNAs with more than 200 nucleotides and lacking protein-coding potential^11^. In recent years, accumulating evidence has indicated that lncRNAs play vital roles in the biological processes of cancer cells^12^, including cell proliferation, migration, invasion, and apoptosis. For example, lncRNA CDKN2BAS promotes metastasis via suppressing miR-153-5p and its upregulation predicts poor prognosis in patients with hepatocellular carcinoma^13^. Linc00472 suppresses cell proliferation and promotes cell apoptosis by sponging miR-196a to upregulate PDCD4 expression in colorectal cancer^14^. B3GALT5-AS1 suppresses liver metastasis via inhibiting miRNA-203 in colon cancer^15^. Similarly, there are also a number of lncRNAs that have been identified as tumor promoters or tumor suppressors in BCa, such as LINC01605^16^ and GAS5^17^. Nonetheless, most other lncRNAs affecting the development of BCa have not been unveiled. In this research, we aim to explore potential lncRNAs related to the progression of BCa.

Accumulating reports have indicated that LINC01116 plays a vital part in many cancers. For all we know, LINC01116 promotes the development of breast cancer by combining with miR-145 to upregulate the expression of ESR1^18^. Besides, LINC01116 also accelerates the progression of epithelial ovarian cancer by means of regulating cell apoptosis^19^. Moreover, LINC01116 knockdown inhibits the progression of oral squamous cell carcinoma by regulating the expression of miR-136^20^. Such broad oncogenic function of LINC01116 in different cancer types aroused our interest in its role in BCa. More importantly, according to data from GEPIA 2 database, we found that LINC01116 has overexpressed in patients with an advanced stages of bladder urothelial carcinoma (BLCA), and the high expression of LINC01116 was associated with a low-survival rate in BLCA patients. Therefore, we planned to further explore the role of LINC01116 in BCa.

As is reported, lncRNAs can regulate the expression of protein-coding genes on the level of transcription or after transcription, which mainly depends on the interaction of lncRNAs with microRNAs (miRNAs)^21^ or RNA-binding proteins^22^ in the cytoplasm or nucleus. In our study, we applied GEPIA 2 and figured out two protein-coding genes (ELK3 and HOXD8) that were most significantly correlated with LINC01116 in BLCA tumors. In addition, ELK3^23^ and HOXD8^24^ were also reported to be involved in the progression of diverse cancer types. Hence, we made an in-depth exploration of the relationship between LINC01116 and ELK3/HOXD8 in BCa.

In summary, we aim to explore the biological role of LINC01116 in BCa and uncover its potential mechanisms in regulating BCa progression. Meanwhile, we first measured the relative expression of LINC01116 in BCa cells. Further, in vitro experiments were employed to elucidate the function as well as the molecular mechanisms of LINC01116 in BCa cells. The findings might be favorable for providing a novel target for the treatment of BCa.

## Materials and methods

### Cell lines and reagent

Human bladder epithelial cell line (SV-HUC-1) and human BCa cell lines (RT-4, 5637, J82, T24) were all available from ATCC Company (Manassas, VA). They were grown in DMEM (Invitrogen, Carlsbad, CA) under 37 °C in 95% air/5% CO_2_ incubator, with 1% antibiotics mixture and 10% FBS as the medium supplements. Totally, 2 mg/ml of Actinomycin D (ActD; Sigma-Aldrich, St. Louis, MO) was acquired for testing mRNA stability.

### Total RNA isolation and quantitative real-time polymerase chain reaction (RT-qPCR)

Total RNA was isolated from the cultured cells employing the Trizol reagent (Invitrogen), and reverse transcribed into cDNA as instructed by the provider (Takara, Shiga, Japan). Gene expression was examined by qPCR using the Prism 7900 HT Fast Real-Time PCR system (Applied Biosystems, Foster City, CA), and defined using the threshold cycle (Ct). The relative level was calculated by 2^−ΔΔCT^ after standardization with reference to GAPDH or U6 expression.

### Plasmid transfection

The designed shRNAs for LINC01116, DKC1, ELK3, and HOXD8, as well as NC-shRNAs (sh-NC) were procured from Ribobio (Guangzhou, China) and transfected into J82 and T24 cells using Lipofectamine^™^3000 (Invitrogen). The miR-3612 mimics/inhibitor and NC mimics/inhibitor, as well as pcDNA3.1/ELK3, pcDNA3.1/HOXD8, and NC-pcDNA3.1 were also produced by Ribobio. After 48 h, transfected cells were reaped.

### Colony formation assay

Clonogenic J82 and T24 cells at the density of 500 cells were seeded into a 6-well plate for 14-day incubation. The clones were initially fixed by 4% paraformaldehyde and then counted manually after staining in 0.1% crystal violet.

### EdU assay

J82 and T24 cells at a density of 5 × 10^4^ were planted into the 96-well plate, and then cultured with EdU medium diluent for 2 h in light of the direction (Ribobio). After fixing and permeabilizing, cells were incubated in DAPI solution, followed by observing by fluorescence microscopy (Olympus, Tokyo, Japan).

### JC-1 assay

Cells in 96-well microplate were centrifuged for 5 min and then loaded with JC-1 dye for 30 min after removing the culture medium. A fluorescent plate reader was applied for the detection of mitochondrial transmembrane potential (ΔΨ*m*). Images were acquired by using fluorescence microscopy.

### Flow cytometry assay

Totally, 2 × 10^5^ J82 and T24 cells were washed in precooled PBS and then collected for detecting cell apoptosis using Annexin V-FITC/PI Apoptosis kit as per user manual (Life Technologies, Carlsbad, CA). Following 15 min of incubation in dark, apoptotic cells were examined by flow cytometer.

### Animal experiment

The 6-week-old male BALB/C nude mice were purchased from Beijing Vital River Laboratory Animal Technology Co. Ltd. (Beijing, China), and used under the approval of the Animal Research Ethics Committee of Qilu Hospital of Shandong University. For xenograft tumor assay, 1 × 10^6^ transfected BCa cells were prepared for subcutaneous injection to mice for 28 days, with tumor volume examined every 4 days. For metastasis assay, 1 × 10^6^ transfected cells were inoculated into nude mice through the tail vein. After sacrificing mice via cervical decapitation, the xenografts dissected from mice were collected for weighing assessment, and the lungs with metastatic nodules were acquired for imaging as well as hematoxylin and eosin (H&E) staining.

### Immunohistochemistry (IHC)

The collected tumor tissues from in vivo xenograft assay were dehydrated after fixing in 4% paraformaldehyde, followed by embedding in paraffin and cutting. Then, sections of 4 μm thick were utilized for IHC assay by use of a specific antibody to Ki-67 or PCNA (Abcam, Cambridge, MA).

### Transwell assay

Totally, 8-mm pore size Transwell chambers were obtained for detecting cell migration, and those with matrigel coating for assessing cell invasion, as requested by the provider (Corning, Corning, NY). The lower chamber was supplied with 100% culture medium, while cells in serum-free medium were plated into the upper chamber for 24 h. After that, cells migrating or invading into the bottom were counted visually via 0.1% crystal violet staining under a light microscope.

### Western blot

J82 and T24 cells in RIPA lysis buffer were loaded on 10% sodium dodecyl sulphate-polyacrylamide gel electrophoresis and transferred onto polyvinylidene fluoride membranes (Millipore, Bedford, MA) at 80 V, followed by treatment with 5% nonfat dry milk. The primary antibodies (1:1000) against E-cadherin, N-cadherin, Vimentin, ELK3, HOXD8, DKC1, and loading control GAPDH, as well as secondary antibodies conjugated with HRP (1: 2000), were all acquired from Abcam. The protein signals were analyzed by an enhanced chemiluminescence detection system (GE Healthcare, Milwaukee, MI) following the guidelines.

### Immunofluorescence (IF)

J82 and T24 cells on coverslips were initially fixed for 10 min, blocked in 3% bovine serum albumin and 0.5% Triton X-100 for 30 min. Then cells were processed with primary antibody (Abcam) against E-cadherin or N-cadherin all night, and then with fluorescence-conjugated secondary antibody at room temperature for 2 h. Nuclei were counter-stained with DAPI, followed by cells visualized by Olympus microscopy.

### Subcellular fraction

After washing in precooled PBS, 1 × 10^6^ J82, and T24 cells were centrifuged at 500 × *g* and then processed with PARIS^™^ Kit (Invitrogen) for subcellular fraction assay. LINC01116 content in both cell cytoplasm and cell nucleus were examined by RT-qPCR and AGE (agarose gel electrophoresis) analyses.

### Fluorescence in situ hybridization

The fixed J82 and T24 cells were rinsed in PBS, dehydrated, and air-dried for cultivating in hybridization buffer with LINC01116-specific RNA fluorescence in situ hybridization (FISH) probe (Ribobio). After DAPI treatment, the stained cell samples were assayed by a fluorescence microscope.

### Luciferase reporter assay

The promoter fragment of ELK3, HOXD8, or LINC01116 was severally inserted into the pGL3 reporter vector after PCR amplification for luciferase assay. The LINC01116 or ELK3 fragment covering the miR-3612 target sites (wild-type or mutant) was inserted into the pmirGLO vector. Following the 48 h of co-transfection to J82 and T24 cells with indicated plasmids, Luciferase Reporter Assay System (Promega, Madison, WI) was used for luciferase activity.

### RNA immunoprecipitation (RIP)

Using the RIP kit, RIP assay in J82 and T24 cells was accomplished as instructed by the supplier (Thermo Fisher Scientific, Waltham, MA). The collected cell lysates were conjugated with the specific antibody to human Ago2, DKC1, or control IgG in magnetic beads, followed by RT-qPCR analysis of the retrieved RNAs.

### RNA pull-down assay

Based on the protocol (Thermo Fisher Scientific), Pierce Magnetic RNA-Protein Pull-Down Kit was applied for RNA pull-down assay. Cell protein extracts were mixed with the specifically biotinylated RNA probes to miR-3612, LINC01116, or ELK3, the magnetic beads with streptavidin were added for 1 h. The mixture of pull-downs was monitored using RT-qPCR or western blot, as needed.

### Chromatin immunoprecipitation (ChIP)

On the basis of an instruction of the ChIP kit (Millipore), ChIP assay in J82 and T24 cells were conducted. After fixing cells for 10 min, the cross-linking between DNA and protein was retained, and then the DNA was randomly fragmented by ultrasonic. The samples were then immunoprecipitated with HOXD8 antibody or IgG antibody (negative control) and then the compound precipitated by magnetic beads. Thereafter, the precipitated fragments were analyzed via RT-qPCR.

### Statistical analyses

The measurement data were exhibited as the mean ± standard deviation of three independent repeats. GraphPad PRISM 6 (GraphPad, San Diego, CA) was employed for processing experimental data by one‐way ANOVA or *t* test, with *p* value below 0.05 defined as the threshold of significant level.

## Results

### LINC01116 knockdown inhibits cell growth in BCa cells

**Fig. 1:**
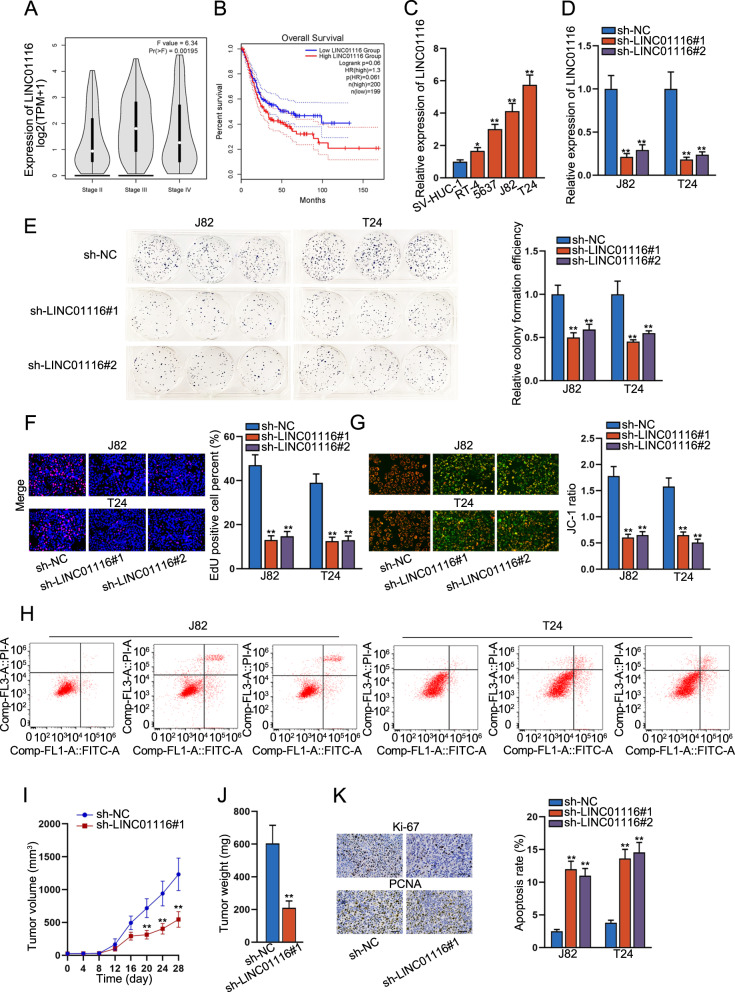
LINC01116 knockdown inhibits cell growth in BCa cell lines. **a** The relative expression of LINC01116 was analyzed in patients with different stages of BCa tissues using the GEPIA 2 bioinformatics website. **b** The overall survival rate was detected by GEPIA 2 bioinformatics website. **c** RT-qPCR was used to detect the expression of LINC01116 in human bladder epithelial immortalized cell line SV-HUC-1 and human BCa cell lines (RT-4, 5637, J82, and T24). **d** RT-qPCR analysis detected LINC01116 expression in J82 and T24 cells transfected with shRNAs targeting LINC01116. **e**, **f** Colony formation assay, and EdU assay tested whether LINC01116 knockdown affects the proliferation ability of BCa cell lines. **g**, **h** JC-1 assay, and flow cytometry analysis revealed the apoptosis rate of J28 and T24 cells transfected with shRNAs targeting LINC01116. **i**, **j** Tumor volume, and tumor weight were measured in response to LINC01116 knockdown. **k** IHC assay detected the staining of Ki67 and PCNA in tumors with or without LINC01116 knockdown. *P < 0.05, **P < 0.01.

To explore the expression of LINC01116 in BCa cell lines, we first analyzed the relative expression of LINC01116 in patients with different stages of BLCA tissues using the GEPIA 2 bioinformatics website (http://gepia2.cancer-pku.cn/#analysis). The results showed that LINC01116 expression increased with the stages progressed in BLCA patients (Fig. 1a, *F* value = 6.34, Pr(>*F*) = 0.00195). Similarly, survival curves were plotted and compared through the aforementioned methods. Comparing to the low LINC01116 level group, the overall survival rate was significantly lower in the high LINC01116 level group (Fig. 1b). Next, we applied RT-qPCR to detect the expression of LINC01116 in human bladder epithelial immortalized cell line SV-HUC-1 and BCa cell lines (RT-4, 5637, J82, and T24). The data indicated that in comparison to SV-HUC-1 cells, LINC01116 expression was markedly boosted in four BCa cells and was highest in J28 and T24 cells (Fig. 1c). So we selected J28 and T24 cells for the following study. To further investigate the functional role of LINC01116 in BCa, we stably silenced the expression of LINC01116 in the selected two cell lines by two shRNAs. The RT-qPCR analysis confirmed that LINC01116 expression was lowered in sh-LINC01116#1 and sh-LINC01116#2 groups compared with the sh-NC group (Fig. 1d). Next, we performed colony formation assay and EdU assay to test whether LINC01116 knockdown affect the proliferation ability of BCa cells. As displayed in Fig. 1e, f, downregulation of LINC01116 suppressed the proliferation ability of J28 and T24 cells. Furthermore, the results of the JC-1 assay and flow cytometry analysis revealed that LINC01116 knockdown increased the apoptosis rate of both J28 and T24 cells (Fig. 1g, h). In addition, we also found that silencing of LINC01116 inhibited the growth of tumors in vivo (Fig. 1i), eventually resulting in lessened tumor size (Fig. S1A) and lowered tumor weight (Fig. 1j). Further, the outcomes of the IHC assay proved that less positivity of Ki67 and PCNA, two proliferation markers, was detected in tumors with depleted LINC01116 (Fig. 1k and S1B). From these findings, we preliminarily judged that LINC01116 plays a promoting role in BCa cell growth.

### LINC01116 inhibits the migration, invasion, and EMT in BCa cells

**Fig. 2:**
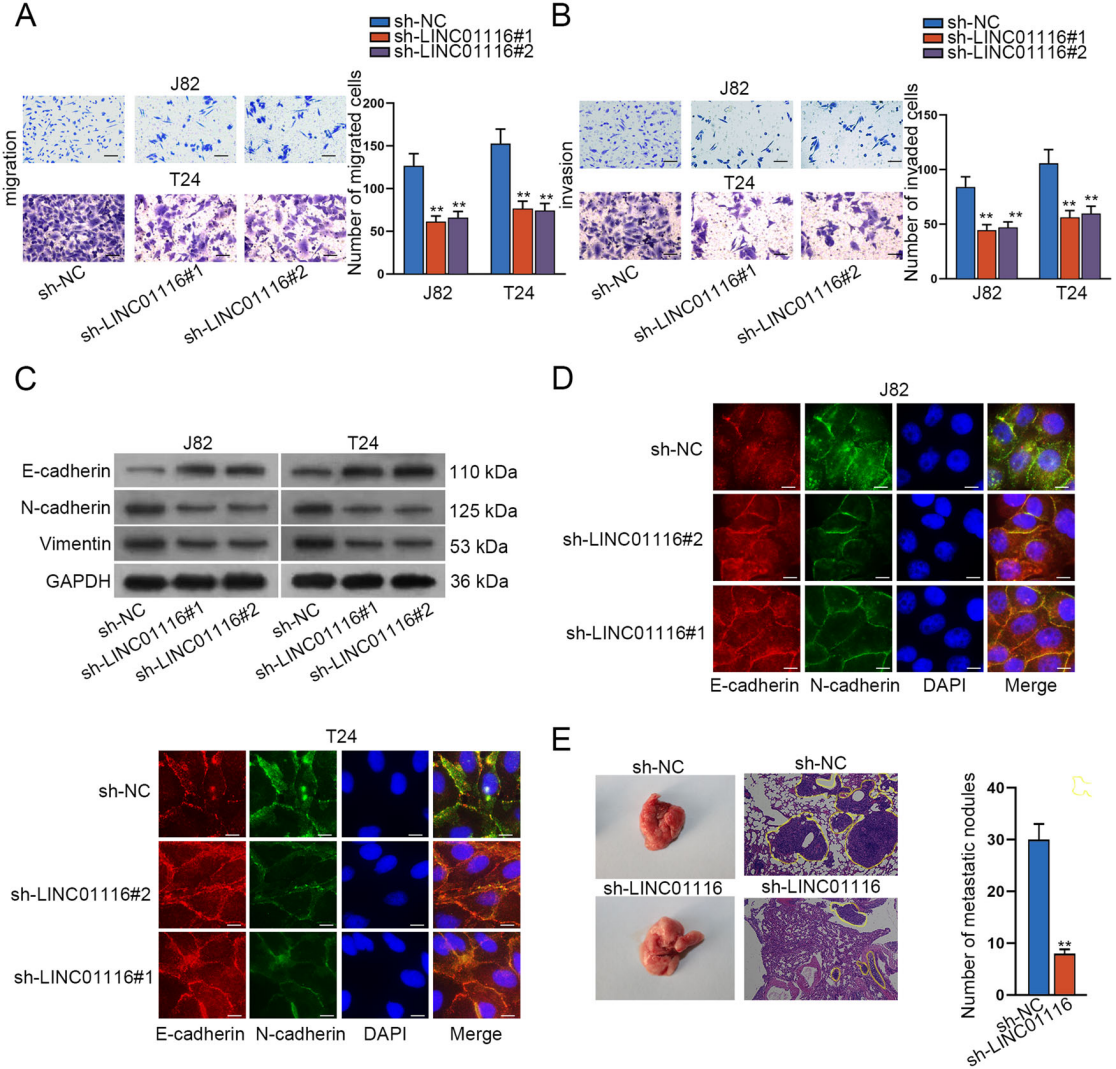
LINC01116 inhibits the migration, invasion, and EMT in BCa cells. **a**, **b** Transwell assays were performed to explore the role of LINC01116 knockdown in BCa cells migration and invasion. **c** E-cadherin, Vimentin, and N-cadherin expression were measured by western blot in J82 and T24 cells transfected with sh-LINC01116#1 and sh-LINC01116#2. **d** IF assay determined the staining of E-cadherin and N-cadherin in J82 and T24 cells with or without LINC01116 silence. **e** The representative image of lungs from the two groups, and HE staining of metastasis nodules in these lungs. *P < 0.05, **P < 0.01.

As we know, cell migration and invasion are crucial to cancer progression and metastasis^25^. Therefore, we performed transwell assays to explore the impact of LINC01116 knockdown on BCa cell migration and invasion. According to Fig. 2a, b, silencing of LINC01116 suppressed the capacity of migration and invasion in BCa cells. Considering that cancer cell metastasis always accompanied by EMT, we then examined the influence of LINC01116 on the EMT process in BCa cells by measuring the expression of E-cadherin, N-cadherin, and Vimentin. The results demonstrated that LINC01116 deficiency obviously increased the expression of E-cadherin in both J82 and T24 cells, while decreased N-cadherin and Vimentin levels (Fig. 2c). Moreover, we also observed the strengthened E-cadherin signals and lessened N-cadherin signals as detected by the IF assay (Fig. 2d). Furthermore, we also performed in vivo metastasis experiments and the outcomes unveiled that the number of metastatic nodules was evidently reduced in the lung of mice injected with LINC01116-silenced BCa cells (Fig. 2e). All these results suggested that silencing of LINC01116 hinders the migration, invasion, and EMT process in BCa cells.

### ELK3 and HOXD8 are modulated by LINC01116 in BCa

**Fig. 3:**
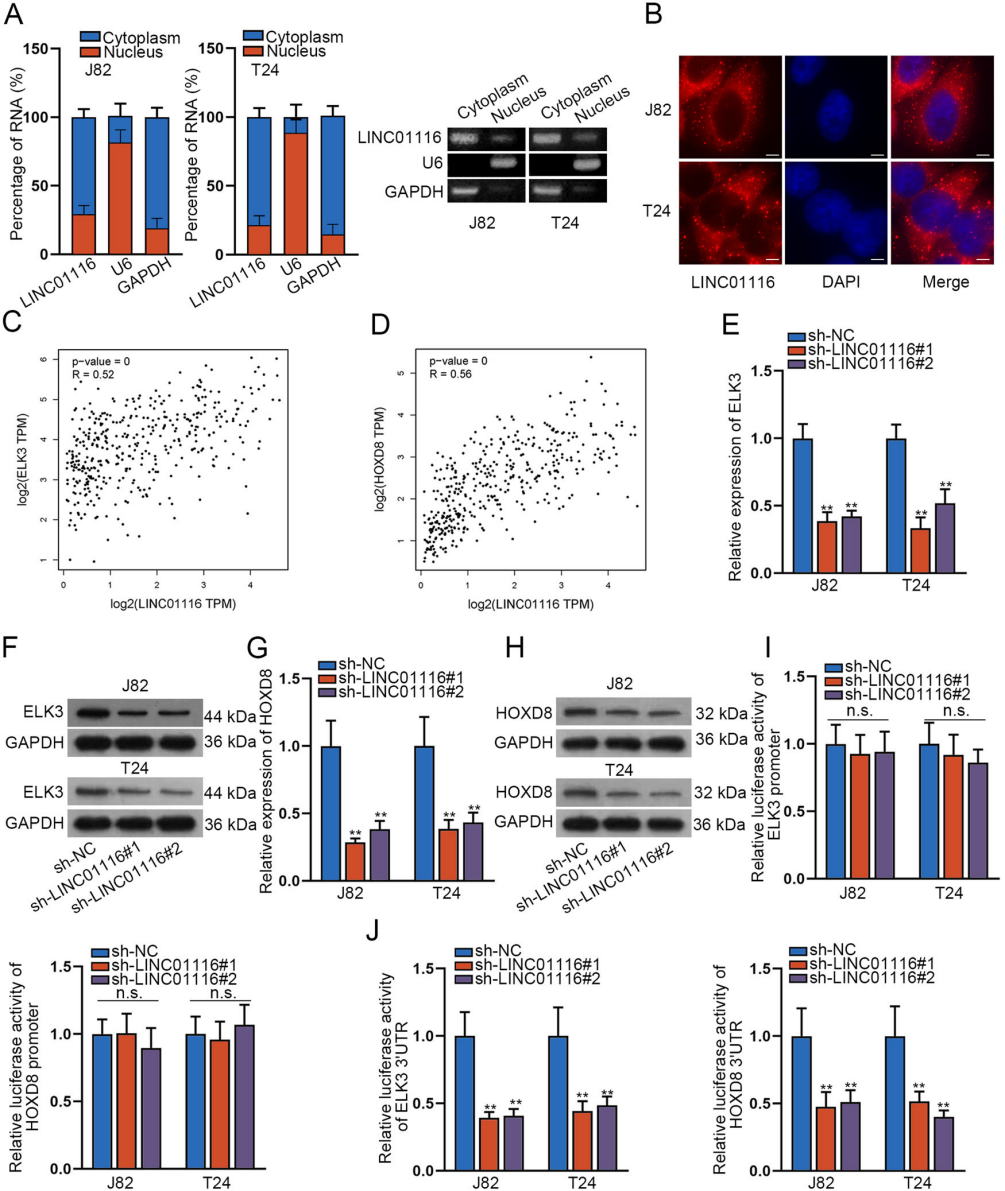
ELK3/HOXD8 and LINC01116 are positive correlations. **a**, **b**. Nuclear separation followed by RT-qPCR and AGE, as well as FISH assay, monitored the subcellular location of LINC01116 in BCa cells. **c**, **d** GEPIA 2 analyzed the relationship between two protein-coding genes (ELK3 and HOXD8) and LINC01116 in BLCA tumors. **e**, **f** RT-qPCR and western blot analyzed the expression of ELK3 in BCa cells under LINC01116 inhibition. **g**, **h** RT-qPCR and western blot analyzed the expression of HOXD8 in BCa cells. **i**, **j** Dual-luciferase reporter assay further validated the combination between ELK3/HOXD8 and LINC01116. *P < 0.05, **P < 0.01.

To determine the regulatory mechanism of LINC01116 in bladder cancer, we then wondered about its subcellular location in BCa cells. As tested by RT-qPCR and AGE following subcellular fraction assay, LINC01116 was mainly distributed in the cytoplasm of BCa cells (Fig. 3a). Consistently, the outcomes of the FISH assay also indicated the high intensity of LINC01116 signals in the cytoplasm (Fig. 3b). Moreover, we used GEPIA 2 analysis (http://gepia2.cancer-pku.cn/#analysis) to find similar genes of LINC01116 in BLCA tumors, and then ELK3 and HOXD8 were predicted as the top two protein-coding genes that were positively correlated with LINC01116 (with the score > 0.5) (Fig. 3c, d). For all we know, previous evidence also reported that ELK3^23^ and HOXD8^26^ played important roles in the development of cancers. Here, we used RT-qPCR and western blot to analyze the expression of ELK3 in BCa cells. The results testified that ELK3 expression was distinctly reduced in both J28 and T24 cells under LINC01116 suppression (Fig. 3e, f). Similarly, the expression of HOXD8 was also apparently reduced in LINC01116-silenced BCa cells (Fig. 3g, h). Based on these data, we speculated ELK3 and HOXD8 were downstream of LINC01116 in BCa. Furthermore, to check the functional relationship between ELK3/HOXD8 and LINC01116, we performed luciferase reporter gene assays. The results disclosed that the relative luciferase activity of ELK3/HOXD8 promoter was no significant altered in J28 and T24 cells transfected with shRNAs targeting LINC01116, whereas the relative luciferase activity of ELK3/HOXD8 3′UTR was remarkably decreased in J28 and T24 cells with LINC01116 inhibition (Fig. 3i, j). All these results suggested the positive correlation between ELK3/HOXD8 and LINC01116 in BCa cells.

### LINC01116 competes with ELK3 in interacting with miR-3612 in the RISCs

**Fig. 4:**
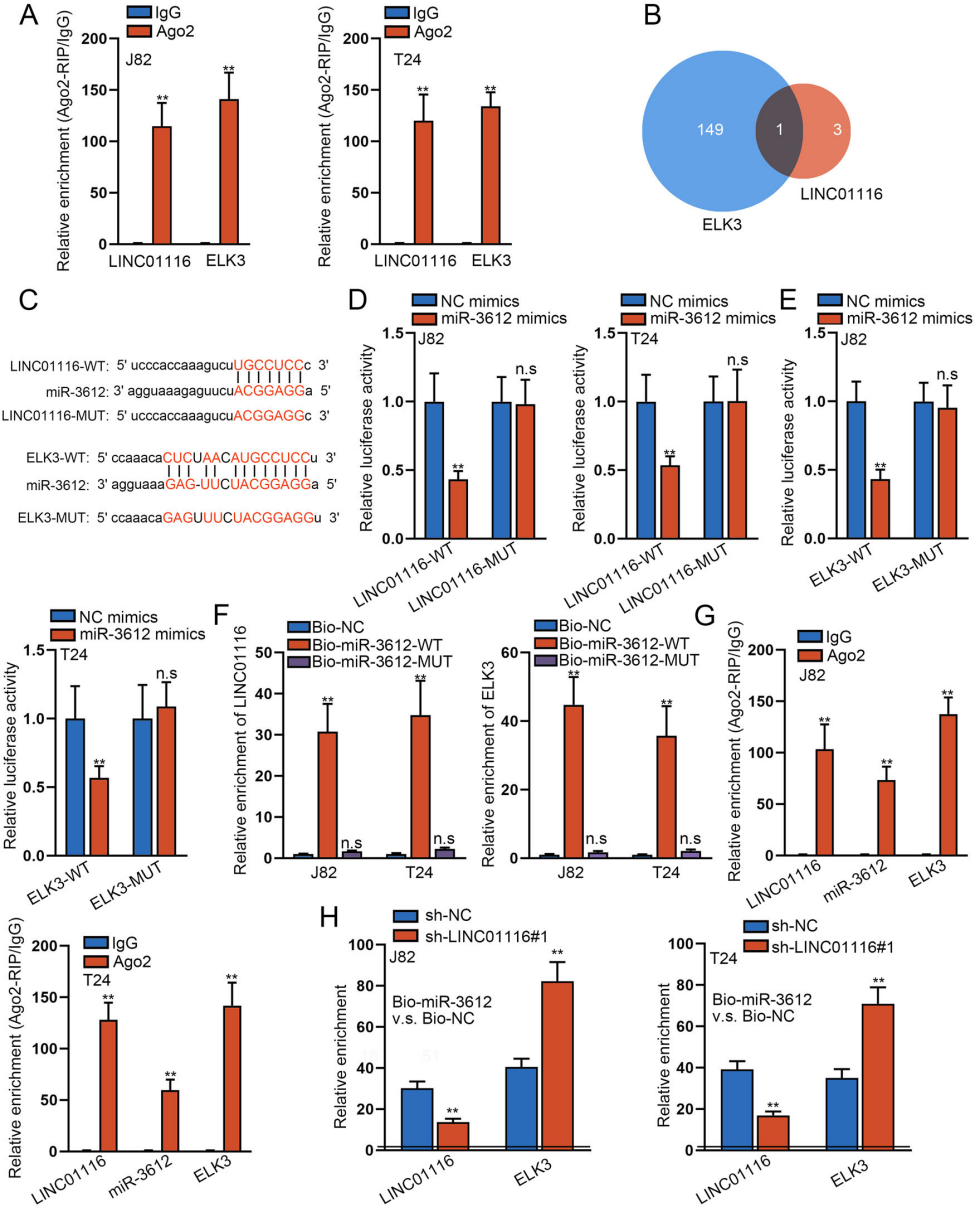
LINC01116, ELK3, and miR-3612 are coexisting in the same RISC. **a** Ago2-RIP assay was performed to prove the existence of LINC01116 and ELK3 in RISCs. **b**, **c** By means of searching on starBase, miR-3612 was predicted to have the potentials to bind with LINC01116 andELK3. **d**, **e** Luciferase reporter gene assays predicted the relationship between miR-3612 and LINC01116/ELK3. **f** RNA pull-down assay further examined the combination between miR-3612 and LINC01116/ELK3. **g** Ago2-RIP assay confirmed the interaction among LINC01116, miR-3612, and ELK3 in RISCs. **h** RNA pull-down assay detected the impact of LINC01116 on the interaction of miR-3612 with ELK3. *P < 0.05, **P < 0.01.

To further prove the relationship between LINC01116 and ELK3, the Ago2-RIP assay was performed. The results confirmed that both LINC01116 and ELK3 were undoubtedly enriched in Ago2 groups (Fig. 4a), indicating that LINC01116 and ELK3 coexisted in the RISCs. By means of searching on starBase, we predicted that miR-3612 could combine with LINC01116 and ELK3 at the same time (Fig. 4b), and the binding sites between them were shown in Fig. 4c. Then we disclosed that miR-3612 was greatly upregulated in J82 and T24 cells after the transfection with miR-3612 mimics (Fig. S1C). Next, luciferase reporter gene assays were proved the combination of miR-3612 and LINC01116/ELK3, since that miR-3612 mimics positively inhibited the luciferase activity of the LINC01116-WT/ELK3-WT, but had no effect on that of the LINC01116-MUT/ELK3-MUT in BCa cells (Fig. 4d, e). Furthermore, RNA pull-down assay results supported that LINC01116 was highly enriched in Bio-miR-3612-WT groups while no such enrichment in Bio-miR-3612-MUT groups (Fig. 4f). Thereafter, the Ago2 RIP assay confirmed that these three RNAs were all obviously enriched in Ago2 groups (Fig. 4g), indicating that their co-existence in RISCs. More importantly, silencing LINC01116 in J82 and T24 cells blocked the binding of miR-3612 to LINC01116 but facilitated the interaction of miR-3612 with ELK3 (Fig. 4h), proving that LINC01116 competed with ELK3 in interacting with miR-3612. Also, we certified that ELK3 expression was markedly downregulated in both J82 and T24 cells after transfection with miR-3612 mimics (Fig. S1D). Moreover, we inhibited ELK3 expression in J82 and T24 cells to probe into its function in BCa (Fig. S2A). As a result, loss of ELK3 led to impaired proliferative ability (Fig. S2B, C), stimulated apoptosis (Fig. S2D), restrained migratory and invasive capacities (Fig. S2E), and hindered EMT (Fig. S2F). Then, to investigate whether LINC01116-affected BCa progression was mediated via miR-3612, rescue experiments were conducted. First, RT-qPCR detected the expression of miR-3612 was evidently decreased in J82 and T24 cells when transfected with miR-3612 inhibitor (Fig. S3A). Next, the outcomes of colony formation and EdU assays displayed that silencing of LINC01116 suppressed the proliferation of BCa cells, and this effect was partly reversed by a miR-3612 inhibitor (Fig. S3B, C). Furthermore, flow cytometry analyzed that miR-3612 inhibitor restored the partial promoting effect of LINC01116 knockdown on cell apoptosis (Fig. S3D). In addition, the results of transwell assays signified that silencing of LINC01116 inhibited the migration and invasion capacity of J82 and T24 cells, which was reversed by a miR-3612 inhibitor to some extent (Fig. S3E, F). Further, western blot analyzed that miR-3612 inhibitor partially counteracted the impact of LINC01116 downregulation on the expression of E-cadherin, N-cadherin, and Vimentin (Fig. S3G). Intriguingly, we verified that miR-3612 inhibition fully reversed the suppression of deficient LINC01116 on ELK3 expression (Fig. S3H). Thus, we concluded that LINC01116 worked as an oncogene promoting the malignant characteristics of BCa cells partly by targeting the miR-3612/ELK3 pathway.

### LINC01116 enhances the stability of HOXD8 through binding to DKC1

**Fig. 5:**
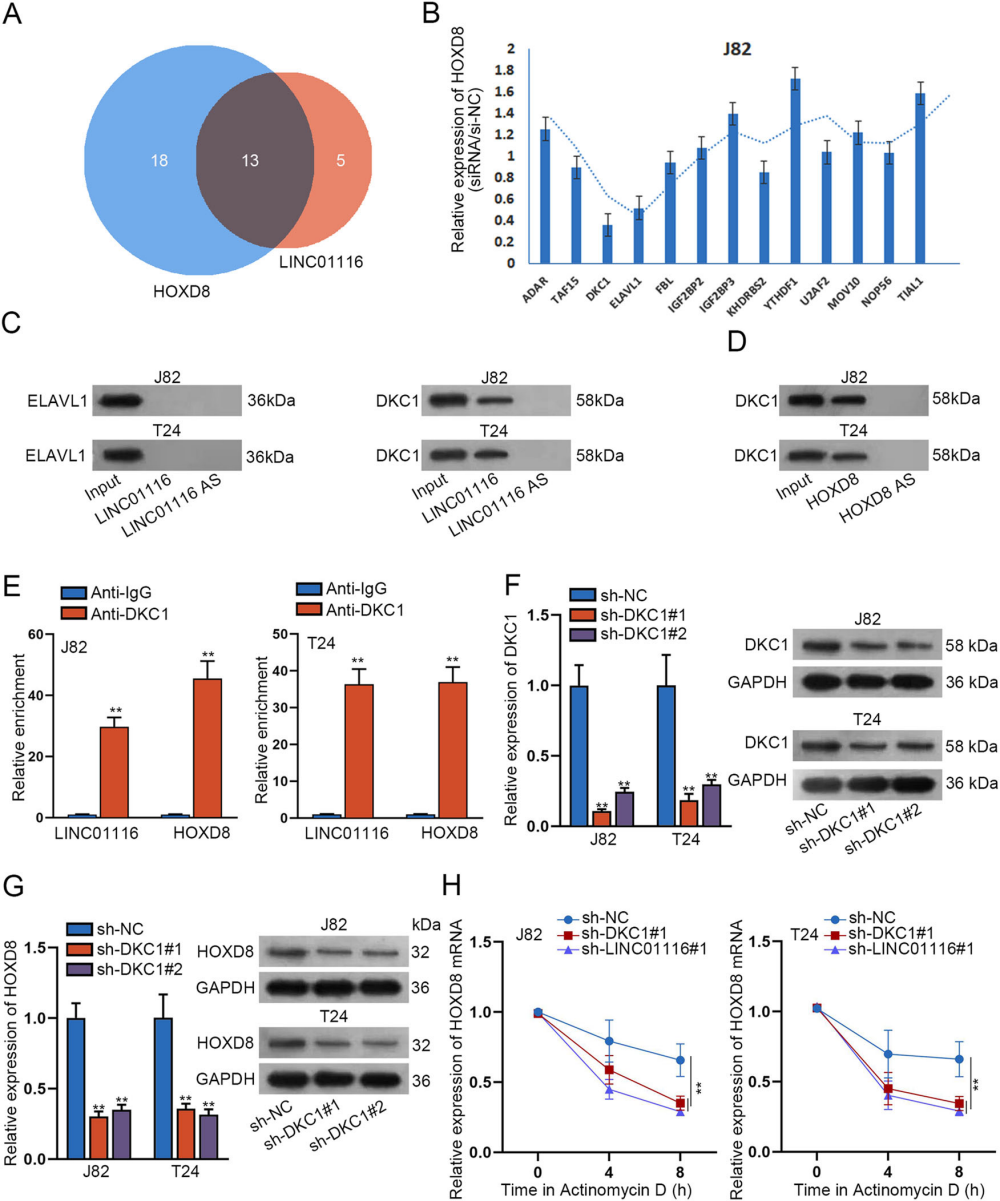
LINC01116 enhances the stability of HOXD8 through binding to DKC1. **a** StarBase software predicted thirteen RBPs shared between HOXD8 and LINC01116. **b** RT-qPCR detected the relative expression of HOXD8 in J28 cells transfected with shRNAs targeting indicated RBPs. **c** RNA pull-down assay tested the interaction of LINC01116 with DKC1 or ELACL1. **d** RNA pull-down assay detected the interaction between HOXD8 and DKC1. **e** RIP assay verified whether LINC01116 or HOXD8 could be enriched in the anti-DKC1 pallet. **f**, **g** RT-qPCR analysis, and western blot analyzed that the expression of DKC1/HOXD8 in J82 and T24 cells transfected with shRNAs against DKC1. **h** RT-qPCR examined the stability of HOXD8 under ActD treatment in J82 and T24 cells transfected sh-LINC01116#1 or sh-DKC1#1. *P < 0.05, **P < 0.01.

Thereafter, we continued to investigate the mechanism whereby LINC01116 regulated HOXD8 in bladder cancer. As we did not find any miRNAs shared by HOXD8 and LINC01116 through analyzing starBase, we then predicted RNA-binding proteins (RBPs) interacting with bothHOXD8 and LINC01116. As reflected in Fig. 5a, there were 13 RBPs that bound with both HOXD8 and LINC01116. Then we used RT-qPCR to detect the changes in the expression of HOXD8 in J28 cells transfected with specific shRNAs targeting the 13 RBPs. The results revealed that the expression of HOXD8 was evidently down-regulated in response to the absence of DKC1 and ELAVL1 (Fig. 5b). Further, the outcomes of RNA pull-down assay indicated that only DKC1 but not ELAVL1 was detected in the complexes pulled down by LINC01116 (Fig. 5c). Besides, the interaction of DKC1 with HOXD8 in BCa cells was also certified by RNA pull-down assays (Fig. 5d). Thus, we proposed DKC1 as the mediator between HOXD8 and LINC01116 in BCa cells. Next, the RIP assay pointed to that both LINC01116 and HOXD8 were apparently enriched in anti-DKC1 groups of J82 and T24 cells (Fig. 5e). In addition, the absence of LINC01116 led to reduced LINC01116 enrichment but elevated HOXD8 concentration in the complexes pulled down by Bio-miR-3612 (Fig. S4A). Furthermore, RT-qPCR and western blot analyzed that the expression of DKC1 was remarkably declined in both J82 and T24 cells transfected with shRNAs targeting DKC1 (Fig. 5f). Consequently, HOXD8 expression was decreased in both J82 and T24 cells with DKC1 depletion (Fig. 5g). To further investigate whether the LINC01116-DKC1 complex could regulate the stability of HOXD8, we respectively transfected shRNAs targeting LINC01116 and DKC1 into J82 and T24 cells treated with ActD. The results demonstrated that the stability of HOXD8 was significantly reduced after the knockdown of LINC01116 or DKC1 compared to the control group (Fig. 5h). Moreover, we found that HOXD8 deficiency impaired cell proliferation, migration, invasion, and EMT, as well as facilitated cell apoptosis in BCa (Fig. S4B–G). In sum, LINC01116 recruited DKC1 to stabilize the mRNA of a tumor-promoter HOXD8 in bladder cancer.

### Upregulation of ELK3/HOXD8 can reverse the inhibiting effect of LINC01116 knockdown on BCa cells

**Fig. 6:**
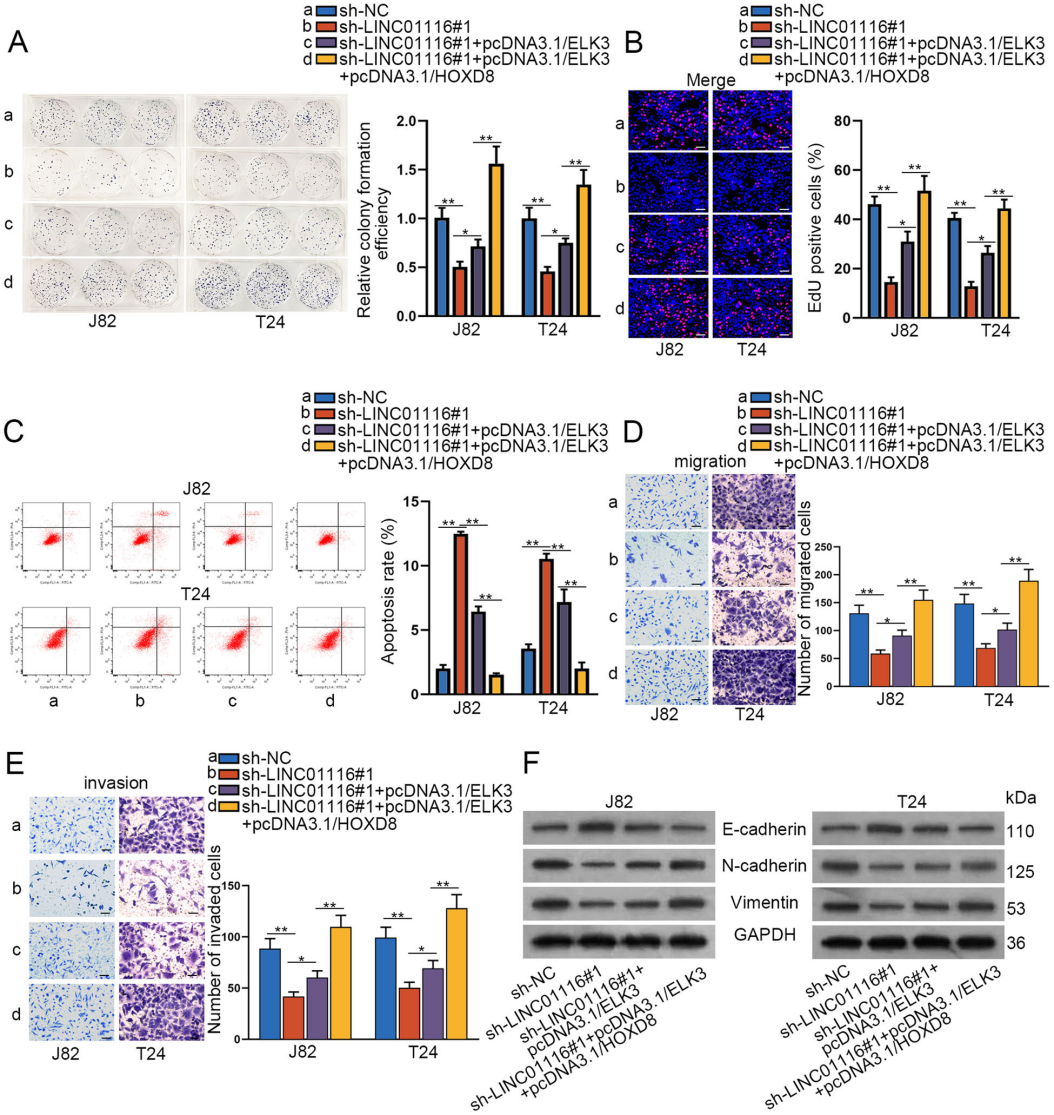
Upregulation of ELK3/HOXD8 can reverse the inhibiting effect of LINC01116 knockdown on BCa cells. Four groups of J82 and T24 cells were involved, including sh-NC, sh-LINC01116#1, sh-LINC01116#1 + pcDNA3.1/ELK3, sh-LINC01116#1 + pcDNA3.1/ELK3 + pcDNA3.1/HOXD8. **a**, **b** Colony formation assay and EdU assay detected the proliferation ability of cells in these four groups. **c** Flow cytometry analysis detected the apoptosis rate of cells in these four groups. **d**, **e** Transwell assays detected the migration and invasion capacity of indicated J28 and T24 cells. **f** Western blot examined the expression of E-cadherin, N-cadherin, and Vimentin in indicated J28 and T24 cells. *P < 0.05, **P < 0.01.

To evaluate whether LINC01116 affected BCa development by ELK3 and HOXD8, rescue assays were carried out. Before that, we testified by RT-qPCR and western blot that the expression levels of HOXD8 and ELK3 were dramatically increased in J28 and T24 cells transfected with pcDNA3.1/HOXD8 and pcDNA3.1/ELK3 (Fig. S4H, I). The results of colony formation assay showed that the relative colony formation efficiency was obviously reduced by LINC01116 knockdown, but was then partly rescued by ELK3 overexpression while absolutely reversed by the promotion of both ELK3 and HOXD8 (Fig. 6a). Similar results were also obtained from EdU assays (Fig. 6b). Next, flow cytometry analysis indicated that ELK3 promotion slightly mitigated the promoting effect of LINC01116 knockdown on cell apoptosis, and this effect was drastically abrogated by ELK3 and HOXD8 promotion (Fig. 6c). Furthermore, co-transfection of pcDNA3.1/ELK3 partially restored the suppressed migration and invasion of J28 and T24 cells transfected with sh-LINC01116#1, while such suppression was completely rescued by co-overexpression of ELK3 and HOXD8 (Fig. 6d, e). Meanwhile, we also applied western blot to detect the expression of E-cadherin, N-cadherin, and Vimentin in J28 and T24 cells. The results revealed that the upregulation of E-cadherin induced by silencing of LINC01116 was abolished by elevated ELK3 in part, and was fully reversed by the co-transfection with pcDNA3.1/ELK3 and pcDNA3.1/HOXD8. The change trends of N-cadherin and Vimentin were opposite (Fig. 6f). All these results indicated that ELK3 and HOXD8 were required in the regulation of LINC01116 on BCa cells.

### HOXD8 serves as a transcription activator of LINC01116

**Fig. 7:**
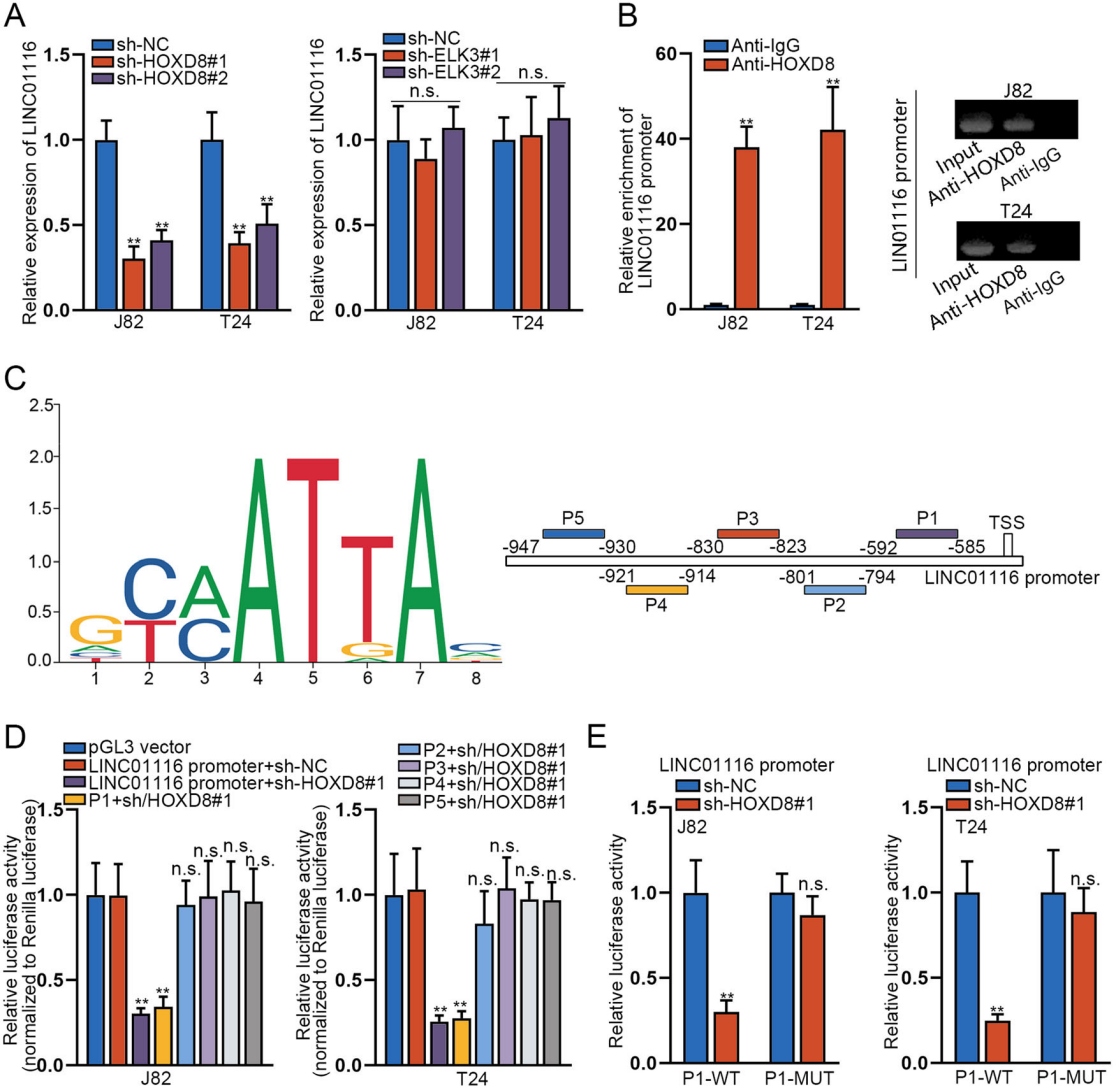
HOXD8 serves as a transcription activator of LINC01116. **a** The expression of LINC01116 was examined in J82 and T24 cells transfected with shRNAs targeting HOXD8 or ELK3 by RT-qPCR. **b** ChIP assay was further conducted to explore the functional relationship between LINC01116 and HOXD8. **c** Five binding sites between LINC01116 promoter and HOXD8 were predicted using JASPAR software. **d** Luciferase reporter gene assays were performed to verify which site in LINC01116 promoter was recognized by HOXD8. **e** Luciferase reporter assays also detected the influence of HOXD8 on the activity of P1. *P < 0.05, **P < 0.01.

Considering that both ELK3 and HOXD8 could serve as transcription factors, here we examined whether they had a feedback regulation on LINC01116. We first examined the impact of HOXD8 or ELK3 on LINC01116 in BCa cells using RT-qPCR. The data confirmed that the expression of LINC01116 was distinctly reduced in J82 and T24 cells in face of HOXD8 inhibition, whereas no significant difference was seen under ELK3 suppression (Fig. 7a), suggesting that HOXD8 might be a transcription factor of LINC01116. Therefore, we applied a ChIP assay to further explore the functional relationship between LINC01116 and HOXD8. The results attested that the LINC01116 promoter was prominently enriched in the anti-HOXD8 group (Fig. 7b). Furthermore, we predicted five binding sites of HOXD8 in the LINC01116 promoter by using JASPAR software (http://jaspar.genereg.net/) (Fig. 7c). To verify which binding site might be responsible for the combination of HOXD8 with LINC01116, we performed luciferase reporter gene assays. The results exhibited that the relative luciferase activity was markedly decreased in LINC01116 promoter + sh-HOXD8#1 group and P1 + sh/HOXD8#1 group, which was seen in both J82 and T24 cells (Fig. 7d), suggesting that P1 was the functional binding site. Moreover, luciferase reporter assays also showed that loss of HOXD8 suppressed the luciferase activity of the P1-WT reporter vector but barely affected that of the P1-MUT reporter vector (Fig. 7e). All these results suggested that HOXD8 was a transcription activator of LINC01116 in BCa.

### LINC01116 functions in the progression of BCa by regulating ELK3 and HOXD8

**Fig. 8:**
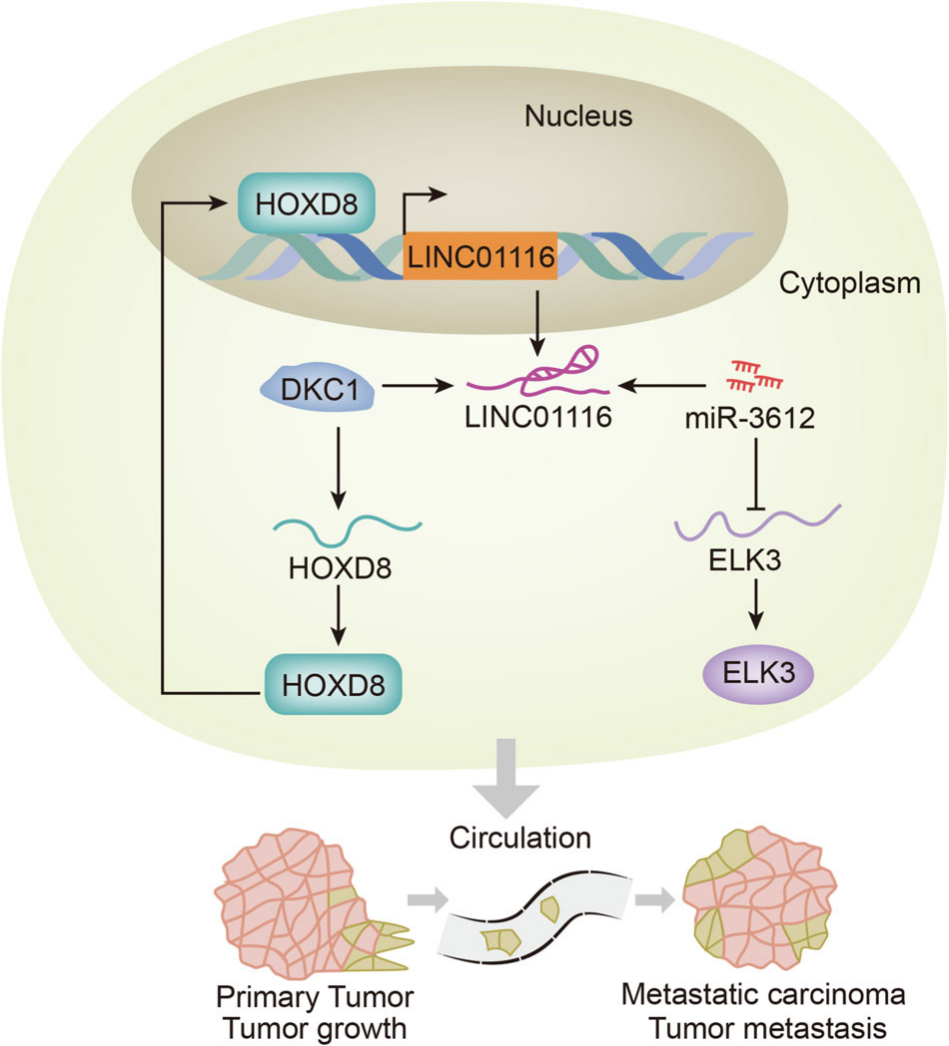
LINC01116 functions in the progression of BCa by regulating ELK3 and HOXD8. The drawing analyzed the two functional pathways of LINC01116-miR-3612-ELK3 and LINC01116-DKC1-HOXD8 in BCa cells. *P < 0.05, **P < 0.01.

As shown in Fig. 8, upregulated LINC01116 could promote tumor growth and metastasis in BCa through two pathways. On the one side, LINC01116 increased the expression of ELK3 by adsorbing miR-3612. On the other side, LINC01116 stabilized HOXD8 by recruiting DKC1. In return, HOXD8 induced the transcriptional activation of LINC01116 in BCa.

## Discussion

Increasing studies have been demonstrated that lncRNAs are brought into the focus in cancers including BCa^27,28^. In other words, lncRNAs with abnormal regulation have been certified to play vital roles in the carcinogenesis and progression of BCa by functioning as tumor suppressors or oncogenes^11,29^. Therefore, the exploration of lncRNAs in BCa may be beneficial to identify more effective biomarkers for the diagnosis and treatment of patients. As reported previously, the tumor-facilitating part of LINC01116 was well-documented in several cancer studies. For instance, LINC01116 promotes the proliferation and migration of osteosarcoma cells by targeting miR-520a-3p and affecting IL6R^30^. LINC01116 enhances the transcriptional activity of MYC to accelerate nasopharyngeal carcinoma progression^31^. Moreover, LINC01116 promotes the progression of osteosarcoma by down-regulating p53 and EZH2^32^. Inconsistent with these findings, our present study verified that LINC01116 was overexpressed in BCa cells, and silencing of LINC01116 significantly inhibited cell proliferation, migration, and invasion, as well as the EMT process in BCa. Collectively, here we also revealed LINC01116 as a promoter of BCa progression.

In the present study, LINC01116 was suggested to be mainly located in the cytoplasm of BCa cells, while cytoplasmic lncRNAs usually function through affecting mRNA stability, translation, or protein modification^33^. Here, we predicted by GEPIA that LINC01116 had a close association with two protein-coding genes ELK3 and HOXD8, and also validated the positive regulation of LINC01116 on them in BCa cells. Further, we verified that the 3′UTR activity of both mRNAs was declined by silenced LINC01116, proving the post-transcriptional regulation of LINC01116 on these two genes. One of the major post-transcriptional mechanisms of lncRNAs is serving as a ceRNA in a regulatory network involving lncRNA, miRNA, and target mRNA^28^. However, we only uncovered miR-3612 as the shared miRNA between LINC01116 and ELK3, but not between LINC01116 and HOXD8. Moreover, the current work proved that ELK3 was a direct target of miR-3612, and LINC01116 could promote the expression of ELK3 by absorbing miR-3612 in BCa cells. According to previous reporters, ELK3 was closely associated with the development of breast cancer^34,35^, liver cancer^36^, colorectal cancer^37^, and prostate cancer^38^. In our study, we confirmed that ELK3 knockdown could markedly suppress cell growth and metastasis in BCa. Interestingly, we also disclosed that the effects of LINC01116 inhibition on BCa cells were only partly rescued by miR-3612 inhibitor, while its impact on ELK3 expression was fully offset under miR-3612 inhibition. These findings meant that LINC01116 modulated BCa progression not only through miR-3612/ELK3 signaling.

Then, we started to explore the way by which LINC01116 affected HOXD8 expression. Eventually, we validated that LINC01116 could stabilize HOXD8 mRNA by recruiting DKC1 in BCa cells. Previously, HOXD8 has been certified to be involved in the progression of cancers, though its role varies with cancer types. Some reports suggest that HOXD8 elicits a repressive function in hepatocellular carcinoma^24^ and colorectal cancer^39,40^, while others argue that HOXD8 also plays as a tumor-promoter in ovarian cancer^41^ and lung cancer^42^. In this study, we proved that inhibiting HOXD8 hampered BCa cell proliferation, migration, invasion, and EMT, revealing HOXD8 as a tumor-facilitator in BCa. Moreover, DCK1 was previously reported to function in the nucleus^43^, but in our study, knockdown of DKC1 could suppress the expression of HOXD8, and LINC01116-DKC1 complex could effectively regulate the stability of HOXD8. Of interest, HOXD8 is suggested as a transcription factor in ovarian cancer^41^. Presently, we demonstrated that HOXD8 served as a transcription activator of LINC01116 in BCa.

In summary, our results revealed that knockdown of LINC01116 could suppress the malignant progression of BCa both in vitro and in vivo, and its role in BCa depended on two pathways. On the one hand, LINC01116 acted as a ceRNA by interacting with miR-3612, thereby reversing the suppressive effects of miR-3612 on ELK3 expression. On the other hand, LINC01116 could enhance the stability of HOXD8 by binding with DKC1. In addition, HOXD8 was a transcription factor that activated LINC01116 in BCa. Thus, LINC01116-miR-3612-ELK3 and LINC01116-DKC1-HOXD8 were suggested as two pathways involving in BCa progression. Significantly, our findings might offer effective targets for anticancer therapies of BCa.

## Supplementary information

Supplementary information

Figure S1

Figure S2

Figure S3

Figure S4
