# Supplementary material for: LINC01106 post-transcriptionally regulates ELK3 and HOXD8 to promote bladder cancer progression
Source: Cell Death Dis. 2020 Dec 12;11(12):1063. doi: 10.1038/s41419-020-03236-9 (PMC7733594; doi:10.1038/s41419-020-03236-9)
Supplement: Supplementary file 1 — Supplementary information [file 41419_2020_3236_MOESM1_ESM.docx]

**Supplementary figure legends**

**Figure S1. LINC01116 inhibition suppresses in vivo tumor growth and ELK3 is targeted by miR-3612.** A. Representative images of tumors derived from BCa cells transfected with sh-NC or sh-LINC01116#1. B. The quantification bar graph of Ki67 and PCNA positivity detected by IHC assays of Figure 1K. C. RT-qPCR tested the overexpression efficiency of miR-3612 in J82 and T24 cells. D. RT-qPCR and western blot examined the expression level of ELK3 in J82 and T24 cells transfected with miR-3612 mimics. *P < 0.05, **P < 0.01.

**Figure S2. ELK3 plays a contributing role in BCa.** A. The knockdown efficiency of ELK3 in J82 and T24 cells was determined by RT-qPCR and western blot. B-E. The impact of silenced ELK3 on the proliferation, apoptosis, migration and invasion of J82 and T24 cells was appropriately assessed by colony formation assay, EdU assay, flow cytometry analysis and transwell assays. F. Western blot detected the level of E-cadherin, N-cadherin and Vimentin in two BCa cells with or without ELK3 inhibition. **P < 0.01.

**Figure S3. The function of LINC01116 in BCa is partially mediated by miR-3612.** A. RT-qPCR detected the expression of miR-3612 in J82 and T24 cells transfected with miR-3612 inhibitor. B-C. Colony formation and EdU assays detected the proliferation of BCa cells transfected with sh-NC, sh-LINC01116#1 or sh-LINC01116#1+miR-3612 inhibitor. D. Flow cytometry analyzed that the changes on cell apoptosis rate between groups of sh-NC, sh-LINC01116#1 or sh-LINC01116#1+miR-3612 inhibitor. E-F. Transwell assays analyzed the migration and invasion of J82 and T24 cells in groups of sh-NC, sh-LINC01116#1 or sh-LINC01116#1+miR-3612 inhibitor. G. Western blot was conducted to examine the expression of E-cadherin, N-cadherin and Vimentin in different groups. H. The impact of LINC01116/miR-3612 on ELK3 expression was examined by RT-qPCR and western blot. *P < 0.05, **P < 0.01.

**Figure S4. LINC01116 affects the expression of a tumor-promoter HOXD8 in BCa by recruiting DKC1.** A. RIP assay evaluated the impact of LINC01116 silence on the interaction of DKC1 with LINC01116 and HOXD8 in BCa cells. B. RT-qPCR and western blot validated the interference efficiency of HOXD8 in J82 and T24 cells. C-F. The changes on the proliferation, apoptosis, migration and invasion of BCa cells under HOXD8 suppression were detected by colony formation and EdU assays, flow cytometry analysis and transwell assay, respectively. G. The level of EMT-related proteins in two BCa cells with or without HOXD8 deficiency was determined by western blot. H-I. RT-qPCR and western blot analyzed the expression of HOXD8 or ELK3 in J28 and T24 cells transfected with pcDNA3.1/HOXD8 or pcDNA3.1/ELK3, separately. *P < 0.05, **P < 0.01.
