# Supplementary figures and images for: LINC01106 post-transcriptionally regulates ELK3 and HOXD8 to promote bladder cancer progression
Source: Cell Death Dis. 2020 Dec 12;11(12):1063. doi: 10.1038/s41419-020-03236-9 (PMC7733594; doi:10.1038/s41419-020-03236-9)

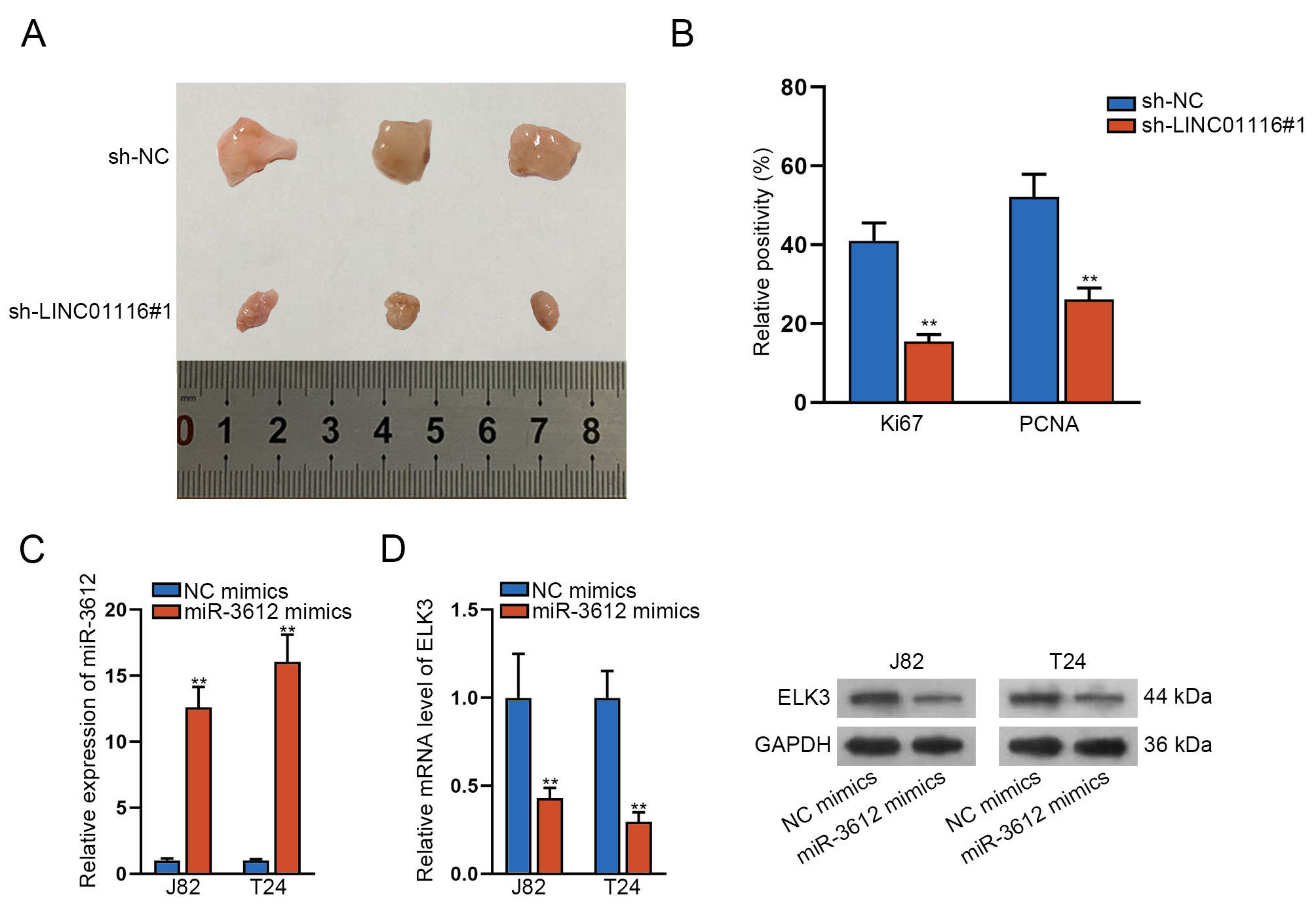

Supplement: Supplementary file 2 — Figure S1 [file 41419_2020_3236_MOESM2_ESM.tif]

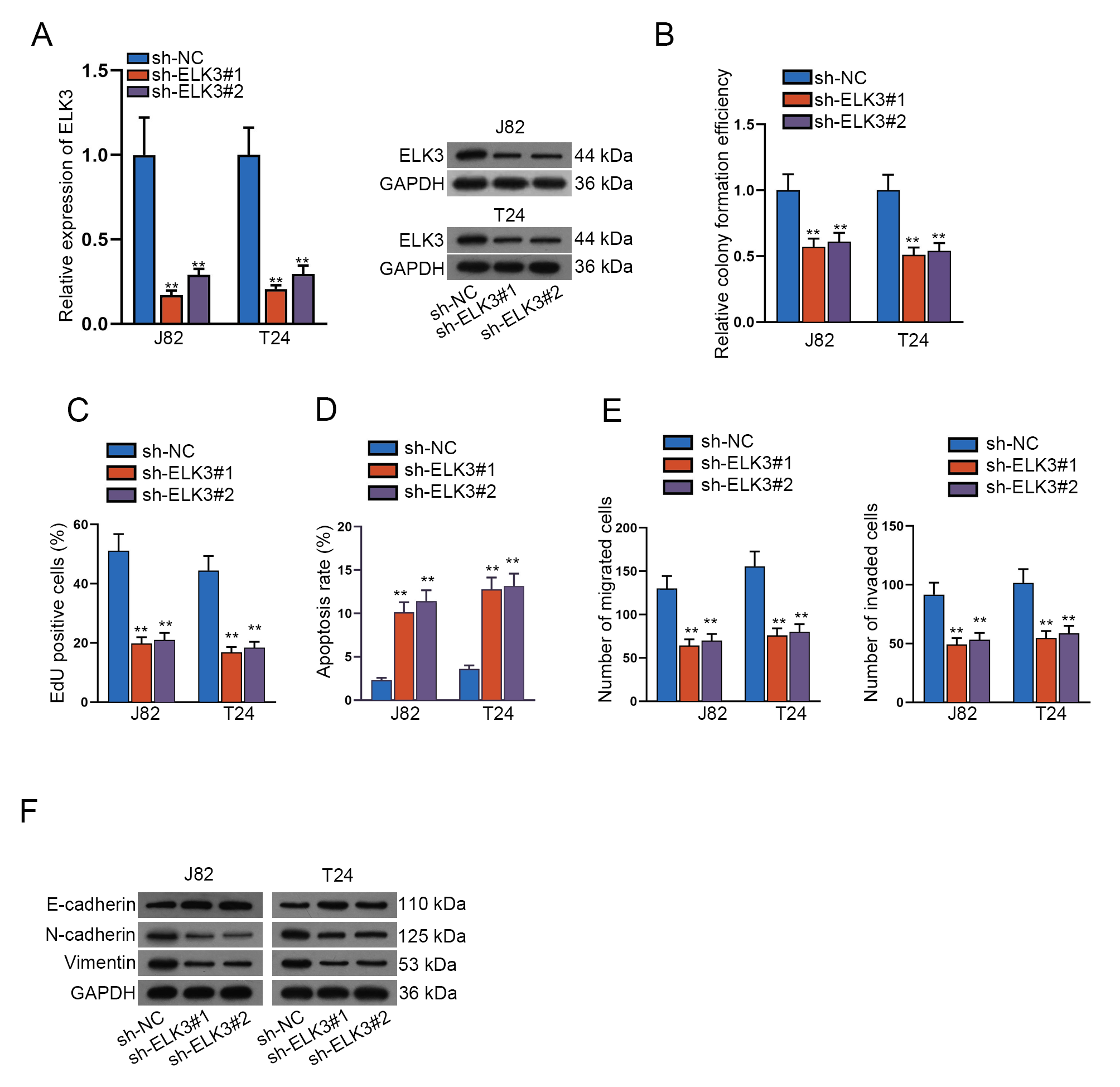

Supplement: Supplementary file 3 — Figure S2 [file 41419_2020_3236_MOESM3_ESM.tif]

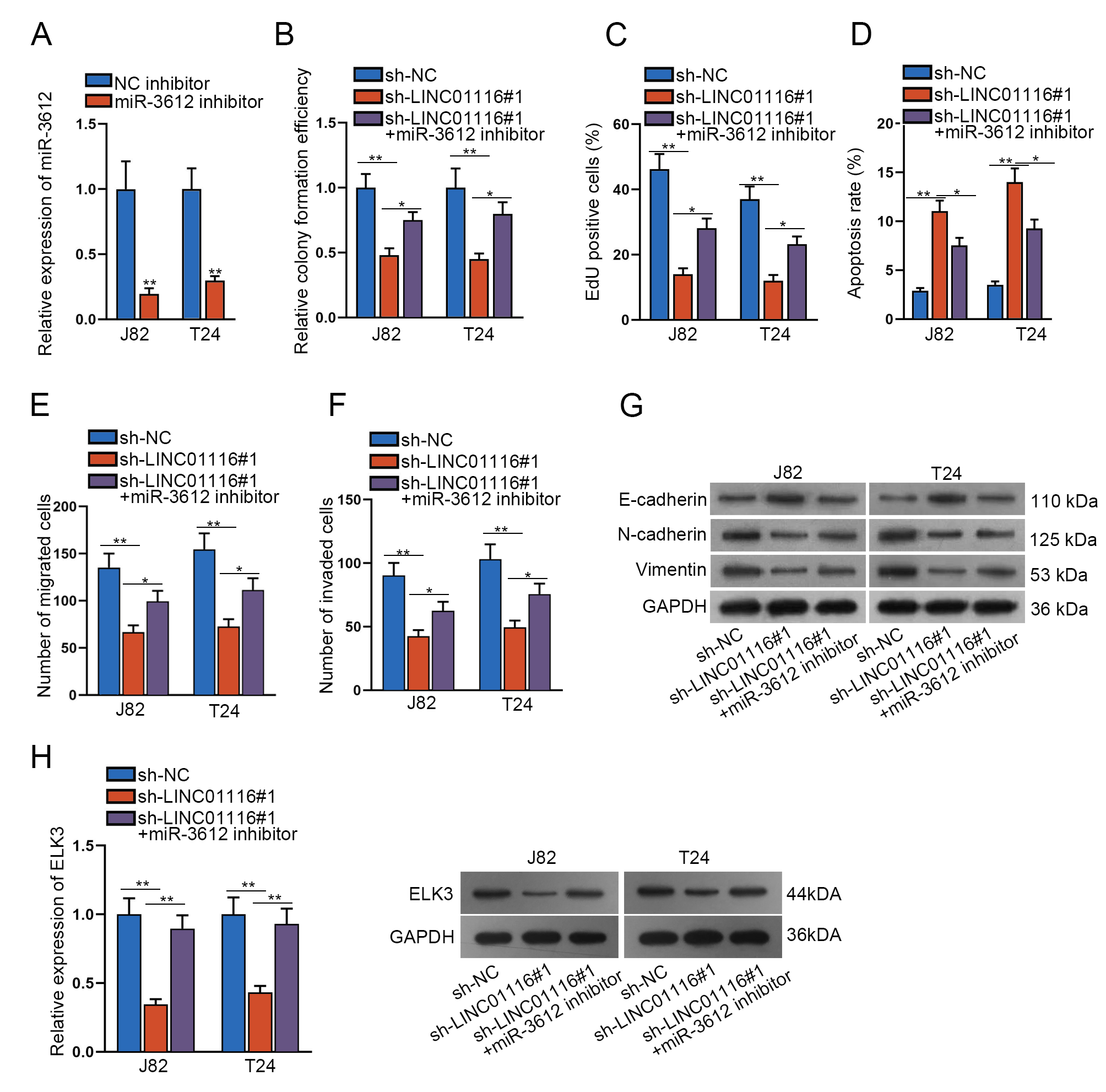

Supplement: Supplementary file 4 — Figure S3 [file 41419_2020_3236_MOESM4_ESM.tif]

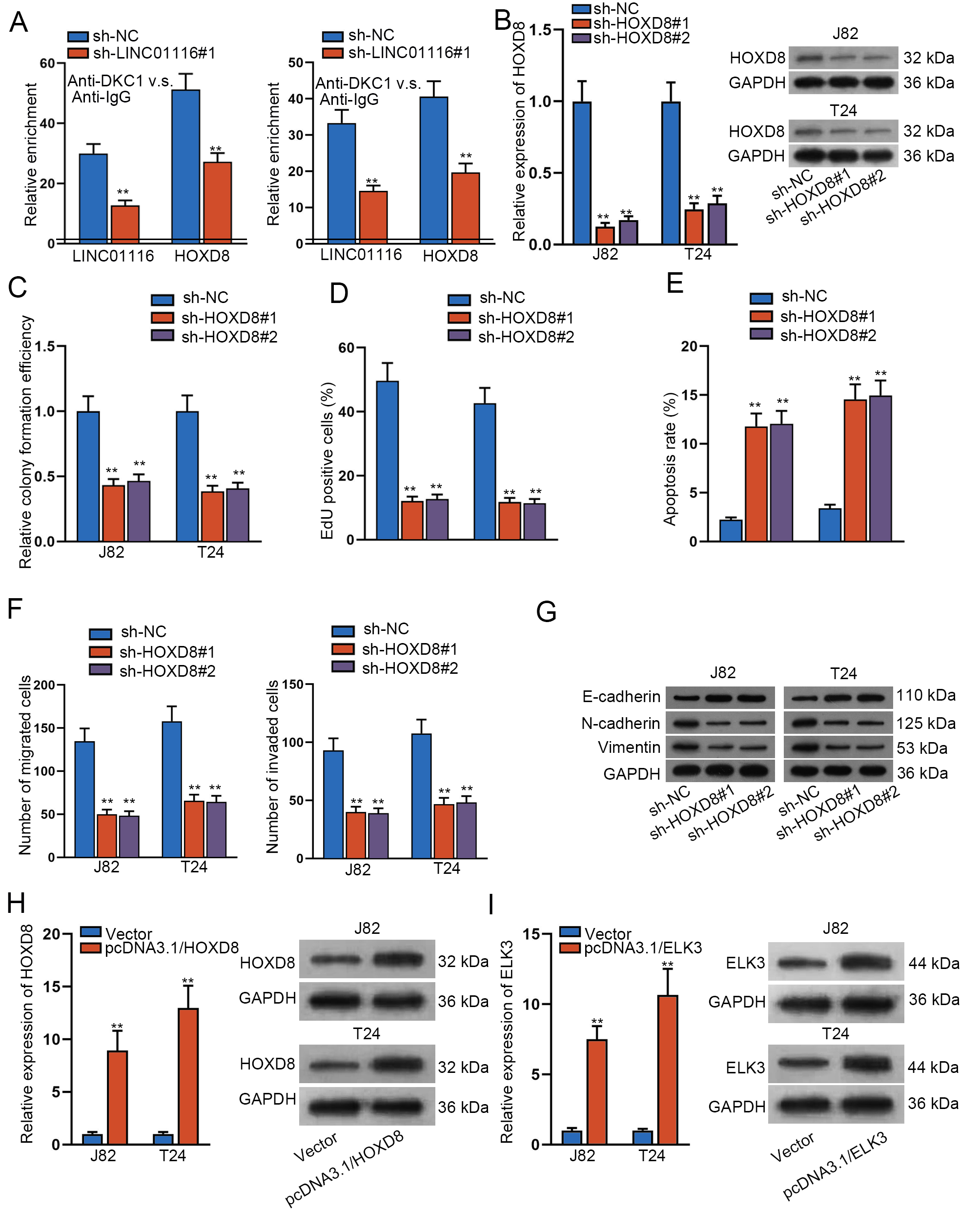

Supplement: Supplementary file 5 — Figure S4 [file 41419_2020_3236_MOESM5_ESM.tif]
